# Modulation of Auditory Cortex Activity in Salicylate‐Induced Tinnitus Rats via Deep Brain Stimulation of the Inferior Colliculus

**DOI:** 10.1002/brb3.70795

**Published:** 2025-09-01

**Authors:** Zeinab Akbarnejad, Kasra Bagherian, Seyed Ali Noorbakhsh, Ali Fathi Jouzdani, Saeid Mahmoudian, Marjan Mirsalehi, Maryam Jafarian, Ronak Mohammadi, Alimohamad Asghari, Abdoreza Asadpour

**Affiliations:** ^1^ ENT and Head and Neck Research Center and Department, The Five Senses Health Institute, School of Medicine Iran University of Medical Sciences Tehran Iran; ^2^ Science Beam Institute Istanbul Turkey; ^3^ Neuroscience and Artificial Intelligence Research Group (NAIRG), Department of Neuroscience, School of Science and Advanced Technologies in Medicine Hamadan University of Medical Sciences Hamadan Iran; ^4^ USERN Office Hamadan University of Medical Sciences Hamadan Iran; ^5^ Brain and Spinal Cord Injury Research Centre, Neuroscience Institute Tehran University of Medical Sciences Tehran Iran; ^6^ Neuroscience Program and Center for Brain and Mind The University of Western Ontario London Ontario Canada; ^7^ Sussex Neuroscience, School of Life Sciences University of Sussex Brighton UK

**Keywords:** spontaneous firing rate, tinnitus, deep brain electrical stimulation, inferior colliculus, auditory cortex

## Abstract

**Background:**

Tinnitus, a self‐reported perceptual disorder, is currently believed to arise from maladaptive plasticity due to reduced sensory input. While deep brain stimulation (DBS) has shown promise in alleviating tinnitus‐related behaviors, its effects on neuronal activity remain unclear. This study aimed to evaluate the spontaneous firing rates (SFRs) of the primary auditory cortex (A1) before and after DBS of the external cortex of the inferior colliculus (ECIC) in a rat model of tinnitus.

**Methods:**

Tinnitus was induced in rats through sodium salicylate injections for 14 consecutive days, while the control group received normal saline injections over the same period. We conducted tinnitus and hearing assessments using the gap pre‐pulse inhibition of the acoustic startle (GPIAS) and pre‐pulse inhibition (PPI) tests. From day 14, both groups underwent DBS of the ECIC and single unit recordings from the A1.

**Results:**

Before ECIC stimulation, A1 neurons in rats with potential tinnitus exhibited significantly higher spontaneous activity compared to controls. Following ECIC stimulation, the SFRs in the group displaying abnormal GPIAS responses significantly decreased, and the difference between the tinnitus and control groups was no longer significant. Additionally, inter‐spike interval (ISI) analysis revealed a higher frequency of short ISIs (<5 ms) in rats with potential tinnitus, which decreased after DBS, aligning with values observed in the control group.

**Conclusion:**

ECIC stimulation effectively modulates A1 hyperactivity, highlighting its role in tinnitus pathophysiology. These findings warrant further research into ECIC's role in tinnitus regulation, which could inform future therapeutic interventions and enhance mathematical models of tinnitus mechanisms.

## Graphical abstract

Deep brain stimulation of the inferior colliculus (ICx) significantly reduces auditory cortex hyperactivity in a rat model of tinnitus. This study highlights ICx stimulation as a promising therapeutic approach for tinnitus‐related neural dysregulation.

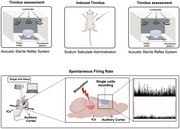

## 1 Introduction

Tinnitus is a self‐reported perceptual disorder characterized by the perception of sound in one or both ears or within the head, despite the absence of an external sound source (Baguley et al. 2013). This common condition affects approximately 10%–20% of the population (Gallus et al. 2015; McCormack et al. 2016) and can negatively impact emotional and social well‐being, often exacerbating psychological symptoms (Hall et al. 2018).

Current research suggests that tinnitus arises from maladaptive plasticity due to reduced sensory input, leading to alterations in homeostatic gain control within the auditory brainstem and cortex (Henton and Tzounopoulos 2021). These disruptions manifest as increased spontaneous neuronal firing, enhanced neural synchrony, and the emergence of thalamocortical dysrhythmia (Henton and Tzounopoulos 2021). Previous studies have documented elevated spontaneous firing rates (SFRs), increased neural synchrony, bursting activity, and tonotopic reorganization in animal models of tinnitus (Eggermont 2003; Noreña 2011; Kaltenbach 2011). In response, various neuromodulation strategies, including brain stimulation techniques, have been explored as potential treatments to suppress abnormal neural hyperactivity (De Ridder et al. 2004; Forogh et al. 2014; Luo et al. 2012). This approach has recently become more popular as a potential treatment for tinnitus (Luo et al. 2012; Cheung and Larson 2010; Shi et al. 2009).

Among these, deep brain stimulation (DBS), an invasive neuromodulation technique, has gained increasing attention for its potential to mitigate tinnitus symptoms (Luo et al. 2012; Cheung and Larson 2010; Shi et al. 2009). Although the precise mechanisms underlying DBS remain unclear, evidence suggests that DBS can modulate pathological neuronal activity patterns, possibly through neuronal silencing (McIntyre and Hahn 2010). Despite its promise, the optimal brain region for neuromodulatory treatment of tinnitus remains uncertain. Previous studies have demonstrated that DBS of the dorsal cochlear nucleus (Luo et al. 2012) and cochlear nucleus reduces tinnitus‐related behaviors in rat models (Aitkin and Phillips 1984).

The inferior colliculus (IC), a key relay station in the ascending auditory pathway, integrates nearly all lemniscal and extra‐lemniscal auditory inputs (Aitkin and Phillips 1984), making it a critical structure in auditory perception. Pathophysiological changes in the IC can significantly alter auditory processing, suggesting its potential role in subjective tinnitus generation (Robertson and Mulders 2012). Within the IC, the external nucleus has been specifically implicated in tinnitus, as animal models exhibit increased spontaneous and bursting activity in this region (Jastreboff 1995). Notably, Smit et al. (Smit et al. 2016) demonstrated that electrical stimulation of the IC normalized the gap: no‐gap ratio in a rat model of tinnitus, providing behavioral evidence of tinnitus suppression. However, the electrophysiological mechanisms underlying inferior colliculus (ECIC) stimulation's effect on tinnitus remain unexplored.

In this study, we aimed to assess the spontaneous activity of the primary auditory cortex (A1) before and after stimulation of the ECIC in a rat model of salicylate (SS)‐induced potential tinnitus. To achieve this, we employed single‐unit recordings as electrophysiological measures to examine changes in spontaneous neuronal activity and compared the results between the group with potential tinnitus and control groups. Consistent with prior research, we observed a significant elevation of spontaneous neuronal activity in A1 for the group with potential tinnitus compared to the control group before the stimulation. However, following DBS of the ECIC, neuronal hyperactivity in the rats with potential tinnitus was markedly reduced, with no significant differences remaining between the two groups.

To our knowledge, this is the first study to report electrophysiological changes in A1 activity following ECIC stimulation in an SS‐induced tinnitus model. These findings provide novel insights into the neuromodulatory effects of ECIC stimulation on tinnitus‐related hyperactivity in the A1.

## 2 Methods

To investigate the effects of DBS of the ECIC on A1 activity, we designed an experiment comparing rats displaying abnormal gap pre‐pulse inhibition (PPI) of the acoustic startle (GPIAS) response with a control group. Behavioral and neural activities were recorded and analyzed to assess the impact of ECIC stimulation. To minimize bias, all electrophysiological and behavioral assessments were done under blinded conditions with respect to the experimental group.

### 2.1 Experimental Design

The study included 14 male Wistar rats aged 9–10 weeks and weighing 250–300 g. Male rats were selected to minimize variability caused by ovarian hormone fluctuations, which significantly influence brain function (Smith et al. 2002; Toffoletto et al. 2014), cognitive performance (Nguyen et al. 2018), emotional regulation (Toffoletto et al. 2014), and susceptibility to neurological disorders (Ysrraelit and Correale 2019). By reducing these potential confounding factors, the use of male rats ensured more consistent and reliable data. Although tinnitus is a complex condition that requires a multidisciplinary assessment, research suggests that treatment outcomes may vary based on sex (Van der Wal et al. 2020). These findings highlight the need for sex‐specific treatment approaches. In this study, male Wistar rats were used to enhance the precision and clarity of experimental results, providing a more controlled evaluation of treatment effects.

We housed the rats in groups of two or three per cage under a 12‐h light/dark cycle, providing unrestricted access to food and water. We maintained environmental conditions at 21–22°C with a humidity level of 50%–55%. All experimental procedures conformed to the guidelines set forth by the National Institutes of Health for the Care and Use of Laboratory Animals (NIH Publication No. 8623, revised 1985) (Health NI of. Guide for the Care and Use of Laboratory Animals 1985) and received approval from the Ethical Committee for Medical Research at Iran University of Medical Sciences (Approval Number: IR.IUMS.REC.1398.1232).

We randomly assigned the animals to either the control or the potential tinnitus group, with seven rats in each. Before administering SS, we conducted a series of behavioral tests, including GPIAS and pre‐pulse inhibition, to establish baseline hearing and tinnitus assessments.

**FIGURE 1:**
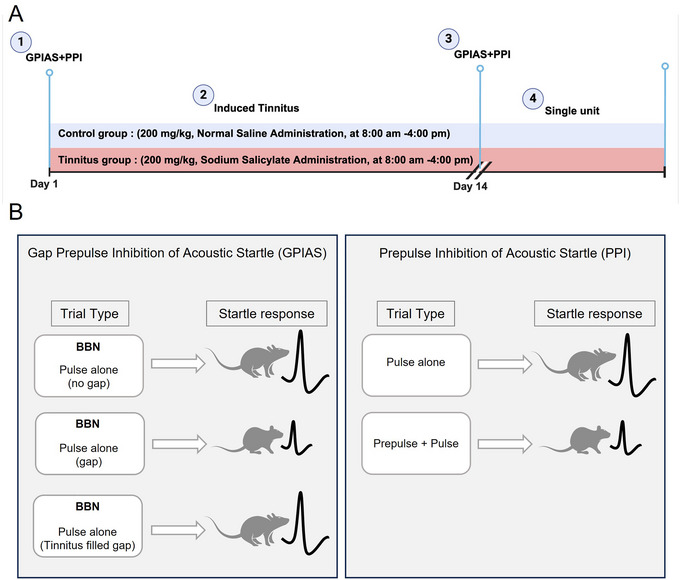
Study protocol and behavioral tests. (A) Timeline of the study protocol. Rats in the tinnitus group received sodium salicylate (200 mg/kg) injections twice daily for 14 days, while the control group received saline injections. Behavioral assessments using gap pre‐pulse inhibition of the acoustic startle (GPIAS) and pre‐pulse inhibition (PPI) were conducted on Day 1 (baseline) and Day 14 to confirm tinnitus induction. Single‐unit recordings from the primary auditory cortex (A1) were performed before and after deep brain stimulation (DBS) of the external nucleus of the inferior colliculus (ECIC). (B) GPIAS and PPI test setups. GPIAS measured startle response modulation with broadband noise (BBN) trials, including gap and no‐gap conditions. PPI evaluated startle inhibition by comparing responses to a startle pulse with or without a pre‐pulse stimulus. These behavioral tests assessed auditory processing and tinnitus‐related deficits.

Following these initial assessments, we administered SS to the tinnitus group and then repeated the behavioral tests to confirm tinnitus induction. Starting on day 14, both groups underwent ECIC DBS and single‐unit recordings from the A1 before and after stimulation to evaluate the effects of ECIC electrical stimulation on A1 spontaneous activity (Figure 1A). Post‐stimulation behavioral assessments (GPIAS and PPI) were not feasible due to technical limitations associated with the electrophysiological recording procedure.

### 2.2 Behavioral Tests for Tinnitus

To assess tinnitus, we performed GPIAS and PPI behavioral tests (Jastreboff 1995) in two stages: before and after SS injection. We used an acoustic startle reflex system (SR‐LAB, San Diego Instruments, USA) to conduct the tests. Each rat was placed in a sound‐attenuating chamber on a piezoelectric platform and secured in a plastic holder.

We measured the downward force generated by the rat's movement on the platform, converting the signal to digital data using SR‐LAB software. A peak‐to‐peak amplitude response was calculated offline. Two ceiling‐mounted loudspeakers delivered both the startle stimulus and a white noise background.

For the GPIAS test, we presented broadband noise (BBN) at 60 dB sound pressure level (SPL), consisting of 16 gap trials and 16 no‐gap trials. A 115 dB white noise pulse lasting 20 ms served as the startle stimulus. Gap trials began 100 ms before the startle bursts and lasted 50 ms with a 1 ms rise and fall time. We set a 20‐s interval between each startle, and each session lasted approximately 25 min.

The PPI test consisted of 15 pulse trials, eight no‐stimulus trials, and 10 prepulse trials. We presented startle stimuli randomly, either alone or following a 60 dB SPL BBN burst prepulse (50 ms duration, 1 ms rise/fall time). The frequency and intensity levels matched those in the GPIAS test, with a 100 ms interval between the prepulse onset and the startle stimulus.

We calculated GPIAS values using the formula (Turner et al. 2006):

(1) $$\mathrm{GPIAS}=\left(1-\frac{\mathrm{Avg}\;\mathrm{gap}}{\mathrm{Avg}\;\mathrm{no}\;\mathrm{gap}}\right)\times 100$$

where “Avg gap” represents the average amplitude during the gap trials and “Avg no gap” represents the average amplitude of the no‐gap trials.

Similarly, PPI values were calculated as (Turner et al. 2006):

(2) $$\mathrm{PPI}=\left(1-\frac{\mathrm{Avg}\;\mathrm{prepulse}}{\mathrm{Avg}\;\mathrm{startle}}\right)\;\times 100$$

where “Avg prepulse” and “Avg startle” refer to the average amplitudes during the prepulse and startle trials, respectively (Figure 1B).

### 2.3 Sodium Salicylate Administration

We prepared a 200 mg/mL solution of SS (Merck, Darmstadt, Germany) by dissolving it in saline. For 14 consecutive days, we administered SS (200 mg/kg) to the potential tinnitus group via intraperitoneal (IP) injection twice daily at 8:00 a.m. and 4:00 p.m. The control group received IP injections of normal saline on the same schedule (Figure 1A).

We used salicylate injections to induce potential tinnitus, as this method significantly increases spontaneous neuronal activity in auditory structures, including the IC and A1. Previous studies have shown that salicylate administration elevates SFRs in the A1 (Duan et al. 2019; Wu et al. 2016) and enhances activity in the IC (Jastreboff and Sasaki 1986; Bauer et al. 2000; Hu et al. 2014), mimicking tinnitus‐related hyperactivity. These effects are associated with altered plasticity due to disrupted gamma‐aminobutyric acid (GABA)‐ergic inhibition in the IC (Bauer et al. 2000; Hu et al. 2014).

Because of the strong connection between the ECIC and A1, we selected this method for its reliability in inducing tinnitus, allowing us to directly assess the effects of DBS.

### 2.4 Surgical Procedure

After 14 days of SS injections, we performed behavioral tests to confirm potential tinnitus‐behavior in the rats. We then conducted the surgical procedure on both groups.

We induced and maintained anesthesia using an intraperitoneal injection of sodium pentobarbital (50 mg/kg). To secure the rats, we used a stereotactic apparatus (Science Beam, Tehran, Iran) with blunt ear and mouth bars. We monitored consciousness by assessing tail and hindlimb reflexes, respiratory patterns (80–100 breaths/min), the absence of voluntary movements, and the lack of obvious whiskering.

### 2.5 Single Unit Recording and Stimulation

**FIGURE 2:**
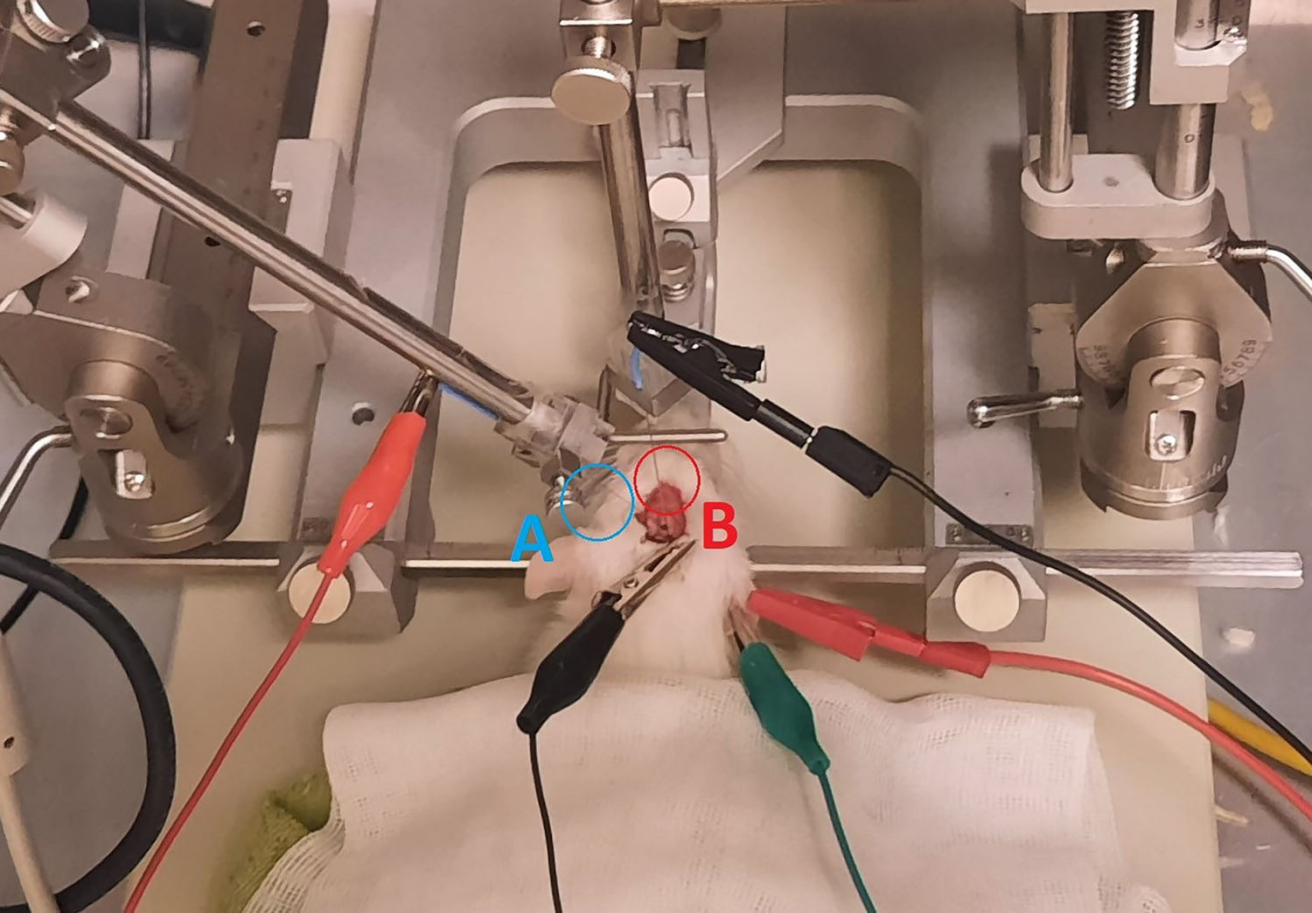
Experimental setup for single‐unit recording and DBS in a rat model. (A) Recording electrode placed in the A1 for measuring spontaneous neuronal activity. (B) Stimulation electrode inserted into the external nucleus of the ECIC for DBS. The stereotactic apparatus ensures precise electrode placement during the procedure. The electrode positions are indicated using arrows.

We used tungsten microelectrodes with an impedance range of 1–10 MΩ (WPI, FL, USA) for recording and stimulation. Stimulation was applied in a monopolar configuration, with the reference electrode placed subcutaneously and the ground electrode attached directly to the pinna. Before electrode insertion, we performed a craniotomy to access the right A1 and right ECIC. Using a stereotaxic atlas (Paxinos and Watson 2006), we placed unilateral electrodes in the right ECIC (Bregma: −7.68, Depth: 4.4, and Interspace: 2.4) for electrical stimulation and in the right A1 (Bregma: −5.04, Depth: 4.4, and Interspace: 6.2) for single‐unit recordings (Figure 2).

We recorded spontaneous activity in the A1 before and after ECIC stimulation to evaluate its effects on A1 neuronal SFRs. we applied monophasic square pulses to the ECIC using a current‐based stimulator (ePulse, Science Beam, Tehran, Iran) via a cable connected to the implanted electrode. Electrical stimulation was delivered using monophasic square pulses (100 Hz pulse frequency, 100 µA pulse amplitude, 60 µs pulse width) via a tungsten electrode for 15 min. We selected these stimulation parameters based on a previous study that successfully induced behavioral modulation in rats with potential tinnitus (Smit et al. 2016). Electrophysiological recordings in the A1 began approximately 5 s after the end of stimulation, allowing sufficient time to avoid stimulation artifacts while capturing early post‐stimulation effects. The recording period lasted 5 min, which was sufficient to assess immediate changes in SFRs following stimulation.

#### 2.5.1 Preprocessing and Extracting the SFR

A1 action potentials were amplified and digitized using an Electro Module (eLab, Science Beam, Tehran, Iran). After recording, we imported the signals into MATLAB (MATLAB 2023) for further analysis.

For preprocessing, a zero‐phase fourth‐order Butterworth bandpass filter (300–6000 Hz) was applied to the data. Spike times were extracted using the mean absolute deviation (MAD) method (Simaan 1997), with the MAD multiplier manually adjusted based on the noise level of the spiking data. After extracting spike times, a spike train was generated and downsampled from 60,000 to 5000 Hz.

We calculated the SFR by applying a sliding window of 1 s as a boxcar kernel using zero‐phase filtering. Additionally, we calculated the inter‐spike intervals (ISIs) from the spike train and estimated the histogram of the ISIs with 5 ms bins for the periods before and after the electrical stimulation.

### 2.6 Statistical Analysis

We determined the sample size using the “equation of resources” approach (Arifin and Zahiruddin 2017), selecting seven rats per group. Statistical analyses for behavioral data were conducted using the Statistical Package for Social Sciences (SPSS V.16; Chicago, USA) (IBM Corp 2007). Normality of the data was assessed through Q–Q plots and the Shapiro–Wilk test (Shapiro and Wilk 1965). Differences in PPI and GPIAS between the two groups were analyzed using an independent samples *t*‐test, while within‐group comparisons were performed using a paired *t*‐test. Figures were generated using GraphPad Prism 10.0 software.

A custom‐written MATLAB program was used to analyze and visualize SFR and ISI histograms, as well as to perform statistical tests on SFR data. To compare SFR between the control and the potential tinnitus groups at each time point, we applied the Kruskal–Wallis test (Kruskal and Wallis 1952), a non‐parametric alternative to the *t*‐test, to account for potential violations of normality. Rank–Biserial correlation (Cureton 1956) was computed as a non‐parametric effect size measure.

For significant time points (*p* < 0.05), effect sizes were categorized into three levels: low (Rank–Biserial correlation < 0.2), medium (0.2 ≤ Rank–Biserial correlation < 0.5), and high (Rank–Biserial Correlation ≥ 0.5). All other results were reported as mean ± standard deviation (SD), with statistical significance set at *p* < 0.05.

For SFR data, we also computed per‐animal means over 5‐min periods before and after stimulation. Normality was assessed using the Kolmogorov–Smirnov test (Smirnov 1948). Depending on distribution, either parametric (unpaired/paired *t*‐tests) (IBM Corp 1908) or non‐parametric (Mann–Whitney *U* test) (Mann and Whitney 1947), Wilcoxon signed‐rank test (Wilcoxon 1945)) comparisons were used. Group comparisons were conducted for the potential tinnitus group versus the control group (before and after stimulation), and within‐group comparisons (before vs. after) were assessed for each group. All tests were two‐tailed, with significance set at *p* < 0.05.

For ISI analysis, we used the two‐sample Kolmogorov–Smirnov test (Smirnov 1948) to compare full ISI distributions between groups, and calculated the proportion of short ISIs (<5 ms) per group. These proportions were compared using chi‐square tests (Pearson 1900). To assess overall activity, total spike counts (i.e., total number of ISIs per rat) were compared before and after stimulation using a paired *t*‐test.

## 3 Results

### 3.1 Chronic Sodium Salicylate Administration Induces a Tinnitus Model

**FIGURE 3:**
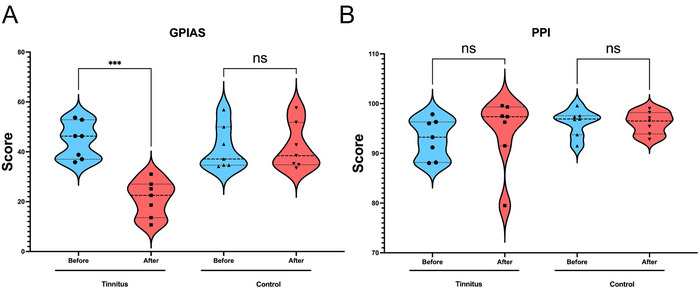
Tinnitus was successfully induced in the tinnitus group without affecting hearing ability. (A) Gap pre‐pulse inhibition of the acoustic startle (GPIAS) scores (%) before and after treatment in the tinnitus and control groups. A significant decrease in GPIAS scores was observed in the tinnitus group after sodium salicylate administration (***p < 0.001), indicating tinnitus induction, while no significant changes were observed in the control group. (B) Pre‐pulse inhibition (PPI) scores (%) before and after treatment in both groups. No significant changes (ns) in PPI scores were observed in either the tinnitus or control groups, confirming that hearing ability remained intact. *N* = 7 per group. Data are represented as violin plots, showing the distribution and individual data points (mean ± SD).

At baseline, the mean GPIAS and PPI values did not differ significantly between the potential tinnitus (44.40 ± 7.35% and 92.97 ± 3.98%, respectively) and control (41.51 ± 8.93% and 96.20 ± 2.69%, respectively) groups (Figure 3A,B). These results confirm that both groups had normal hearing before the experiment.

Following 14 days of SS injections, the GPIAS score of the potential tinnitus group significantly decreased to 21.20 ± 7.33% compared to baseline (*p* = 0.018), indicating tinnitus induction (Figure 3A). In contrast, PPI values remained unchanged (42.01 ± 9.335%, *p* = 0.701), confirming that SS administration did not impair hearing ability (Figure 3B).

In the control group, neither GPIAS (42.01 ± 9.335%) nor PPI (96.13 ± 2.243%) changed significantly after 14 days (Figure 3A, B), demonstrating the stability of auditory function in these animals.

### 3.2 ECIC Stimulation Modulates Spontaneous Neural Firing Rate in A1

**FIGURE 4:**
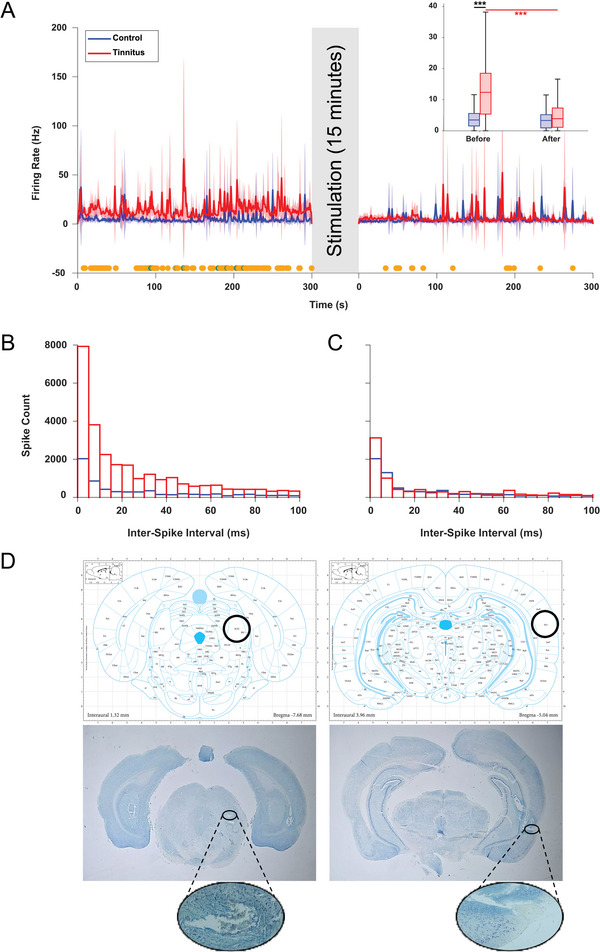
ECIC stimulation reduced tinnitus‐related AC hyperactivity and confirmed accurate electrode placement. (A) The firing rate chart before and after electrical stimulation indicates significant differences between the individual firing rates in the control and tinnitus groups. Dark green dots (low effect size, Rank–Biserial correlation < 0.2), orange dots (medium effect size, 0.2 ≤ Rank–Biserial correlation < 0.5), and teal dots (high effect sizes, Rank–Biserial correlation ≥ 0.5). Shaded areas: 95% confidence intervals (CI); Inset: Boxplots summarize mean SFRs across animals for each group before and after ECIC stimulation (***p < 0.001). (B,C) Interspike interval histograms of the tinnitus and control group before stimulation and after stimulation, depicting the firing intervals in (A). (D) ECIC (left) and A1 (right) representative photomicrograph showing histological localization. The top image presents an anatomical reference adapted from the Paxinos and Watson atlas (Paxinos and Watson 2007). The bottom left (right) panel shows the electrode site in the ECIC (A1), with an elliptical inset highlighting a magnified view of the electrode tract. The bottom images were acquired using a stereo microscope and an Olympus BX51 microscope (DP72 camera, 10× objective). The electrode sites are marked with black circles.

We next examined the effects of ECIC stimulation on the SFR of A1 neurons across the control group and the potential tinnitus group. Before stimulation, the potential tinnitus group exhibited significantly elevated SFRs compared to controls (mean ± SD: 15.11 ± 2.64 Hz vs. 5.17 ± 1.31 Hz; *p* < 0.001, unpaired *t*‐test), confirming a hyperactive neuronal state consistent with tinnitus pathophysiology ECIC (Figure 4A).

Following ECIC stimulation, the mean SFR in the potential tinnitus group significantly decreased to 6.07 ± 2.03 Hz (*p* < 0.001, paired *t*‐test), representing a substantial suppression of aberrant firing. In contrast, the control group showed no significant change after stimulation (before: 5.17 ± 1.31 Hz; after: 4.84 ± 1.42 Hz; *p* = 0.67). Furthermore, post‐stimulation SFRs were statistically comparable between the two groups (*p* = 0.21) (Figure 4A). These findings suggest that ECIC stimulation effectively reduces abnormal A1 hyperactivity. Effect size analysis showed that the most significant time points exhibited a medium effect size, indicating a moderate reduction in SFR differences between the potential tinnitus and control groups after stimulation.

To further investigate neuronal activity, we analyzed ISI distributions. Before stimulation, the potential tinnitus group displayed a higher frequency of short ISIs (<5 ms) compared to the control group (Figure 4B), indicating increased neuronal firing. After DBS of the ECIC, the ISI distribution in the potential tinnitus group shifted toward values more comparable to the control group (Figure 4C).

Kolmogorov–Smirnov tests confirmed significant differences between ISI distributions in rats with potential tinnitus versus control rats, both before and after stimulation (*p* < 0.0001).

The proportion of short ISIs (<5 ms) remained higher in animals with potential tinnitus before (24.89%) and after (24.43%) stimulation than in controls (19.86% and 19.93%, respectively), with both comparisons yielding significant chi‐square values (Before: χ^2^ = 9959; After: χ^2^ = 5151; *p* < 0.0001). Notably, the total spike count in the potential tinnitus decreased substantially after stimulation (*p* < 0.0001, paired *t*‐test), indicating that while the relative proportion of short ISIs remained higher, the absolute number of rapid‐firing events was reduced, consistent with suppressed hyperactivity.

This suppression of pathological hyperactivity in both SFR and ISI measures following stimulation strongly supports the therapeutic potential of ECIC DBS for tinnitus, demonstrating its ability to restore neuronal homeostasis in the auditory cortex. Additionally, histological verification confirmed that the electrodes were correctly positioned in both the ECIC and A1, ensuring precise stimulation and recording locations (Figure 4D).

## 4 Discussion

This study provides the first electrophysiological evidence of tinnitus suppression following ECIC stimulation in a rat model of tinnitus. Our findings revealed that SS administration induced potential tinnitus‐like behavior in rats, which was associated with elevated SFRs and shorter ISIs, indicating increased basal neuronal activity compared to controls. Notably, ECIC stimulation significantly reduced spontaneous activity in rats with potential tinnitus, an effect not observed in the control group. These results align with Smith et al.’s study (Smit et al. 2016), which reported a significant reduction in tinnitus‐related behaviors following ECIC stimulation. While Smith et al. used 16 kHz noise exposure to induce tinnitus and assessed perception using GPIAS and PPI, we directly recorded neuronal activity post‐DBS.

To induce a persistent pattern of chronic potential tinnitus state, we administered SS injections for 14 days, as even a single dose of SS is known to increase spontaneous neuronal activity in the A1 (Duan et al. 2019). Our findings support previous studies indicating that SS‐induced potential tinnitus is associated with increased spontaneous activity in the IC and altered plasticity due to reduced GABAergic inhibitory control (Duan et al. 2019; Wu et al. 2016; Jastreboff and Sasaki 1986; Bauer et al. 2000; Hu et al. 2014). Given the connectivity between the ECIC and A1, heightened ECIC activity is expected to contribute to increased neuronal firing in the A1 of rats with potential tinnitus.

Our results indicate that ECIC stimulation modulates altered plasticity potentially linked to tinnitus‐like perception by reducing spontaneous activity within the ECIC, which in turn decreases neuronal firing in higher auditory areas, such as the A1. One possible explanation for this effect is residual inhibition (RI), a phenomenon in which tinnitus is temporarily suppressed following a stimulus such as magnetic, electrical, or acoustic stimulation (Savastano 2004; Mahmoudian et al. 2015). RI effects typically last from seconds to minutes, though in some cases, suppression can persist for hours or even a full day (Hazell and Wood 1981). Tinnitus is strongly associated with increased spontaneous neuronal activity, and previous research by Galazyuk et al. demonstrated a close link between RI and neural response suppression (Galazyuk et al. 2019). Their findings suggest that a reduction in hyperactive spontaneous neuronal firing may underlie the tinnitus suppression observed during RI. Additionally, their study highlights the critical role of metabotropic glutamate receptors in driving RI mechanisms (Galazyuk et al. 2019).

DBS appears to modulate pathological neuronal activity patterns, potentially through inhibitory effects on hyperactive neurons (McIntyre and Hahn 2010). One hypothesis suggests that high‐frequency stimulation induces functional effects similar to a lesion (Herrington and Cheng 2016), effectively suppressing aberrant neuronal firing. Like acoustic stimulation, ECIC stimulation follows a bottom‐up approach in auditory processing (Zhang 2013). Although tinnitus suppression via acoustic and electrical stimulation may operate through different mechanisms, both methods likely disrupt neural correlates of tinnitus along auditory and non‐auditory pathways. Evidence suggests that tinnitus‐related signals can be modulated by neuroplasticity or may compete with acoustic stimulation (Schechter and Henry 2002). Electrical stimulation of the ECIC may directly modulate or disrupt hyperactivity within the IC, activating neuronal circuits involved in tinnitus perception. Additionally, ECIC stimulation could enhance the IC's modulatory role within ascending auditory pathways, further influencing tinnitus‐related activity. In this context, the midbrain auditory implant has been introduced as an alternative neuroprosthetic option for people with profound hearing loss who are not eligible for a cochlear implant, particularly those with cochlear nerve defects or advanced cochlear ossification (Lim et al. 2009). Stimulation of the inferior colliculus forms the basis of this neural prosthesis, facilitating direct interaction between central auditory pathways in the midbrain (Lim et al. 2009). Over the past two decades, both surface and penetrating stimulation strategies have been explored, highlighting the IC as a viable alternative to the cochlear nucleus for restoring auditory perception in selected patients. Notably, surface stimulation using Med‐El ABI arrays (Colletti et al. 2007) and penetrating stimulation via cochlear DBS array (Lenarz et al. 2006; Lenarz et al. 2007) have demonstrated that IC‐targeted activation can induce reliable auditory sensations without significant adverse effects. These advances—rooted in anatomical accessibility, tonotopic organization, and multisensory integration in the IC—highlight its potential as a central site for hearing prostheses (Lim et al. 2009). The transition from early experimental efforts to promising clinical results represents significant advances in electrode design, surgical targeting, and our understanding of midbrain auditory coding.

In a recent systematic review, Basner et al. (2024) evaluated the available evidence on the safety and effectiveness of DBS for treating refractory tinnitus. The review included three studies that encompassed seven patients who received DBS targeting four subcortical structures: the caudate nucleus, nucleus accumbens (NAc), a ventrally located subregion of the anterior limb in the internal capsule (vALIC), and the medial geniculate body (MGB). Among these, stimulation of the caudate was the most frequently reported, showing moderate improvement in tinnitus functional index (TFI) scores. Single cases involving NAc/vALIC and MGB stimulation demonstrated more substantial symptom relief. DBS may be a suitable neuromodulatory strategy for managing treatment‐resistant subjective tinnitus. Preliminary data from small‐scale studies support the emerging safety and efficacy of this approach (Basner et al. 2024).

While we did not identify the laminar origin of the recorded units or classify neurons into inhibitory and excitatory types, our methodology aligns with previous electrophysiological studies in the A1 that focused on population‐level responses. Prior studies, for example, Jaramillo and Zador (2011), Niwa et al. (2012), and Otazu et al. (2009) successfully interpreted cortical dynamics without resolving fine‐grained cellular details. These findings support the validity of our approach and suggest that valuable insights into auditory cortical processing can be obtained through non‐laminar, non‐cell‐type recordings.

Our findings demonstrate the potential of ECIC stimulation to reduce tinnitus‐related hyperactivity using commonly applied stimulation parameters in animal models of various diseases (Smit et al. 2016; Temel et al. 2005). However, the optimal stimulation parameters remain undetermined, and the temporal extent of their effects is yet to be elucidated through longer‐term recordings. These limitations, which also affect clinical DBS applications, highlight the need for further research to refine stimulation protocols for tinnitus treatment. Moreover, this study exclusively used male rats to minimize the variability introduced by hormonal fluctuations. However, given the evidence that tinnitus pathophysiology and treatment responses may vary by sex (Van der Wal et al. 2020), future studies should incorporate both male and female subjects to ensure broader applicability of findings. Another limitation concerns the absence of layer‐specific or cell‐type‐resolved analyses, which may limit the ability to accurately interpret the spatial and functional organization of neuronal activation patterns. Furthermore, incorporating post‐stimulation auditory threshold assessments in future studies could help elucidate stimulation‐induced changes in auditory sensitivity. Additionally, previous mathematical models of tinnitus have incorporated various frameworks, for example, cortical and subcortical networks with inhibitory and excitatory connections (Schaette and Kempter 2009), Bayesian predictive coding (Sedley et al. 2016), and dynamic causal modeling (Tsai et al. 2018). However, these models have largely overlooked the role of the ECIC. Given its ability to modulate tinnitus‐related A1 hyperactivity, future computational models should integrate ECIC dynamics to improve theoretical understanding and guide therapeutic interventions.

## 5 Conclusions

This study demonstrated that 14 consecutive days of salicylate administration induced behavioral signs consistent with tinnitus‐like perception in rats, accompanied by a significant increase in spontaneous firing rates in the A1. Notably, DBS of the ECIC effectively reduced spontaneous activity in a model exhibiting behavioral signs consistent with chronic tinnitus, suggesting its potential as a neuromodulatory intervention. These findings contribute to a better understanding of the role of the ECIC and its projections to the A1 in both the etiology and treatment of tinnitus, facilitating the way for future research on ECIC‐targeted therapies.

## Author Contributions

**Zeinab Akbarnejad**: project administration, methodology, writing original draft, investigation. **Kasra Bagherian**: investigation, writing original draft**. Seyed Ali Noorbakhsh**: provision and maintenance of the single unit recording system, together with the services. **Ali Fathi Jouzdani**: writing original draft, formal analysis, software, writing original draft. **Saeid Mahmoudian**: methodology, data curation. **Marjan Mirsalehi**: project administration, writing original draft. **Maryam Jafarian**: preparation of histological samples and administration. **Ronak Mohammadi**: visualization, writing original draft. **Alimohamad Asghari**: conceptualization, project administration, validation. **Abdoreza Asadpour**: conceptualization, formal analysis, software, supervision, writing—review and editing. All authors have read and approved the final version of the manuscript.

## Ethics Statement

All experimental procedures were approved by the Ethical Committee for Medical Research of the Iran University of Medical Sciences (Ethical approval number: IR.IUMS.REC.1398.1232).

## Peer Review

The peer review history for this article is available at https://publons.com/publon/10.1002/brb3.70795

## Data Availability

The MATLAB scripts used for spike detection, firing rate analysis, and inter‐spike interval comparisons, along with a link to sample analyzed data, are available at https://github.com/asadpouretal/ECIC‐Tinnitus‐DBS. The repository includes code to reproduce key analyses and generate primary figures in the study. The fully analyzed data and raw spike data are not included in the repository due to size constraints, but are available from the corresponding authors upon reasonable request.
